# Engineering photonics solutions for COVID-19

**DOI:** 10.1063/5.0021270

**Published:** 2020-09-01

**Authors:** Maria Soler, Alexis Scholtz, Rene Zeto, Andrea M. Armani

**Affiliations:** 1 Nanobiosensors and Bioanalytical Applications Group (NanoB2A), Catalan Institute of Nanoscience and Nanotechnology (ICN2), CSIC, BIST and CIBER-BBN, Barcelona, Spain; 2 Department of Biomedical Engineering, University of Southern California, Los Angeles, California 90089, USA; 3 Mork Family Department of Chemical Engineering and Materials Science, University of Southern California, Los Angeles, California 90089, USA

## Abstract

As the impact of COVID-19 on society became apparent, the engineering and scientific community recognized the need for innovative solutions. Two potential roadmaps emerged: developing short-term solutions to address the immediate needs of the healthcare communities and developing mid/long-term solutions to eliminate the over-arching threat. However, in a truly global effort, researchers from all backgrounds came together in tackling this challenge. Short-term efforts have focused on re-purposing existing technologies and leveraging additive manufacturing techniques to address shortages in personal protective equipment and disinfection. More basic research efforts with mid-term and long-term impact have emphasized developing novel diagnostics and accelerating vaccines. As a foundational technology, photonics has contributed directly and indirectly to all efforts. This perspective will provide an overview of the critical role that the photonics field has played in efforts to combat the immediate COVID-19 pandemic as well as how the photonics community could anticipate contributing to future pandemics of this nature.

## I. INTRODUCTION

**FIG. 1.**
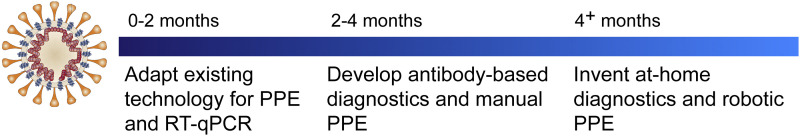
Overview of the different technological solutions being pursued to address COVID-19.

From microscopy^1–3^ to optical communications,^4–6^ optical technologies permeate nearly every aspect of society. Therefore, when confronted with the challenges of a global pandemic,^7–12^ it is not surprising that many solutions have been found in photonic devices and developed by optical engineers. Although research and development efforts have been hindered by work from home conditions and manufacturing has been delayed by shortages in the supply chain,^13^ photonics researchers and companies have made significant contributions to diagnostics and personal protective equipment (PPE) by both adapting existing systems and inventing new technologies (Fig. 1).^8,10,14^

The short-term solutions have focused on inventing easily manufacturable biomedical devices and on addressing the global shortages in personal protective equipment (PPE) to reduce spread, particularly in healthcare settings. This work has included the development of fabric face masks, 3D printable face shields, and respirators for healthcare workers. While optical technologies played only a supporting role in the PPE fabrication efforts, they directly contributed to PPE re-use. Specifically, numerous approaches that leverage the ability of ultraviolet-C (UV-C) or UV germicidal irradiation (UVGI) to serve as a disinfection method were developed.^15–17^ In the face of the pandemic, many countries accelerated approvals of various technologies or granted emergency use authorization. As a result, these safety measures were available to the healthcare community within weeks. In addition, many existing technologies were either re-configured or adapted to more directly address COVID-19 needs.

The mid-term and long-term solutions emphasized developing methods for tracking and accelerating pharmacological solutions. Accurate tracking of infected individuals to reduce and to contain COVID-19 spread requires a combination of software and hardware (diagnostics). While software solutions were quickly launched, accurate diagnostics have been more challenging to deploy, in part, because of the numerous fundamental questions about the pathophysiology of COVID-19 that remain unanswered. As a result, many diagnostics, including optical diagnostics, are finding an immediate use in understanding the nature of the disease.^18,19^

In parallel, researchers are also pursuing the development of therapeutics and vaccines. While outside of the scope of this perspective, numerous potential strategies are being investigated with the hope that one will be proven effective quickly.^20–33^ Additionally, it is notable that the fundamental approach to vaccine and therapeutic trials has also been re-envisioned, greatly accelerating the potential timeline of availability.

This perspective will focus on the role of optics in healthcare, discussing technologies for disinfection and diagnostics. For both topics, a brief background is followed by a more in-depth discussion on recent innovations and their impact to society. We end by discussing the future prospects and open questions in the field.

## II. EXISTING HEALTHCARE MONITORING TECHNOLOGIES

Since the time of Hippocrates, diagnosis of disease has played a key role in medicine and healthcare. Initial approaches relied on palpation and analyzing physical symptoms, such as temperature. As technology progressed, physicians and scientists developed more advanced diagnostic methods, for example, analyzing the cell shape and color of red blood cells. In the modern era, medical diagnostics has transitioned from the cellular to the molecular level, and it typically relies on an integrated transducer platform to facilitate device fabrication. Additionally, with the development of instruments such as MRI, NMR, and CT, imaging is no longer limited to *ex vivo* and *in vitro* methods.

Integrated diagnostic platforms for detecting either molecular or protein indicators of disease based on electrical, mechanical, and optical transduction mechanisms have been demonstrated. All three types of sensors have been used extensively in research applications. For example, mechanical sensor arrays based on cantilevers have been used to weigh individual cancer cells, investigating the efficacy of different therapeutics. Electrical sensors based on nanopore arrays have been used to analyze and sequence DNA, and optical sensors based on plasmonics have been used to understand antibody–antigen binding reactions. However, the majority of commercialized diagnostic systems that are utilized in medical settings for disease diagnosis are based on optical technologies due to the simplicity of the optical signal readout and compatibility with a wide range of sample types. For example, optical biosensing methods have increased the precision and accuracy of disease diagnosis, optical detectors have enabled disease progression monitoring, and laser-based treatments and therapeutics have improved the therapeutic efficacy and shortened recovery times.^34–38^ In the context of COVID-19, many existing technologies have been rapidly re-configured and applied for both diagnostics in a healthcare setting and at-home monitoring of disease progression.

**FIG. 2.**
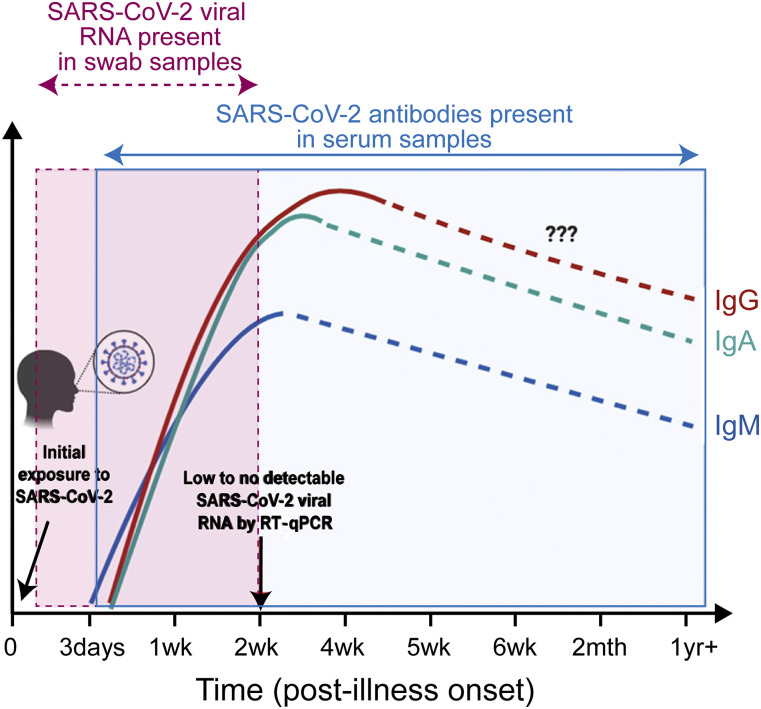
The concentration of RNA and antibodies indicative of a SARS-CoV-2 infection varies with time since infection. Adapted with permission from Lee *et al.*, Front. Immunol. **11**, 879 (2020). Copyright 2020 Author(s), licensed under a Creative Commons Attribution 4.0 License.

Most molecular diagnostic platforms for healthcare settings rely on detecting either the RNA or the DNA of the pathogen^39–43^ or the immune system response to the pathogen (antibody).^38,44–47^ Therefore, before a diagnostic can be developed, it is necessary to either obtain and sequence the RNA or DNA or identify the antibody generating the response. From a diagnostic perspective, it is more straightforward to perform the former because it directly detects the virus. Additionally, RNA or DNA methods detect the active circulating pathogen, potentially in pre-symptomatic patients (Fig. 2).^18,27,39,48^ In the case of COVID-19 and other highly contagious pathogens, this ability is critical as it allows preventative measures to reduce transmission to be taken, potentially preventing or containing outbreaks.^12,18^

In contrast, an antibody-based diagnostic infers the presence of the virus through the person’s immune system response.^49^ Therefore, it is an indirect indicator of infection, and it is much more susceptible to incorrect findings. Additionally, it requires knowledge of the antibody that is specific to the pathogen. Importantly, because antibody-based techniques will only show positive diagnosis after the immune system has responded, these methods are unable to detect pre-symptomatic infections (Fig. 2). Therefore, in the case of COVID-19, it is not surprising that the development and widespread adoption of an RNA-based test occurred well in advance of an antibody-based assay.^14,19,50–53^

**FIG. 3.**
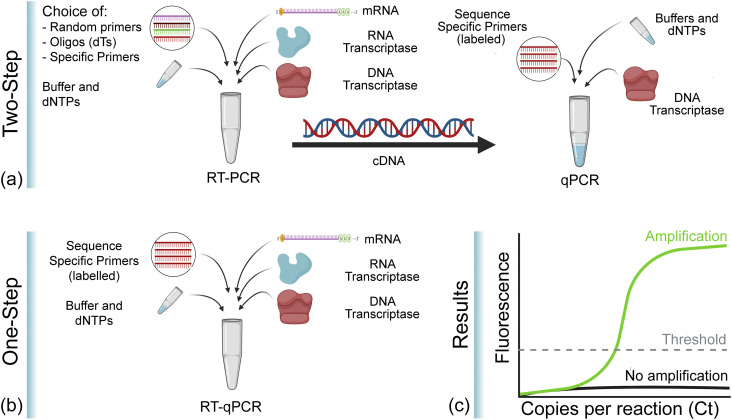
Comparison of PCR methods for diagnosis of COVID-19. (a) Two-step PCR. First, RNA is converted to DNA through RT-PCR. Then, DNA is quantified using qPCR. This approach allows for optimization of both reactions, but it is more time-consuming than a one-step process. (b) One-step PCR. RT-PCR and qPCR are performed in the same vial in parallel. This approach is faster, but the two reactions are not optimized. (c) Cartoon of the type of data that are generated and analyzed. Images created with Biorender.com.

Once the RNA sequence was established, conventional DNA methods, namely, reverse transcription-polymerase chain reaction (RT-PCR), in combination with existing sample handling and processing protocols, were leveraged to convert the viral RNA located in the nasal swab sample to DNA and to amplify the DNA concentration to detectable levels using RT-PCR (Fig. 3).^32,48,54,55^ While RT-PCR increases the sample concentration, detection is typically performed using quantitative-PCR (qPCR), which is a fluorescence-based sensing method.^42,43^ It is important to note that RT-PCR and qPCR are distinctly different steps in this process; however, qPCR and RT-PCR can be performed simultaneously, allowing the concentration to be measured in real time. This approach is often called real-time RT-PCR (or RT-qPCR) in shorthand.

As a fluorescent-based detection technology that relies primarily on the visible spectrum, qPCR (and RT-qPCR) has benefited greatly from numerous advances in optical technology, ranging from optical sources to detectors as well as novel fluorophores. The reduction in footprint and increased lifetime of LEDs relative to conventional bulbs has allowed for a reduction in system size and lowered maintenance and operating costs. For high performing systems, the availability of narrow linewidth sources and high sensitivity detectors covering a wide spectral range has enabled signal multiplexing. When combined with robotic samplers for automated handling, a single instrument can analyze hundreds of samples per hour with low false positive/false negative rates. Although the technology required for PCR technologies has improved significantly, because PCR detects RNA and DNA directly from the virus, PCR technologies can only detect active infections, and not past infections once the patient recovers.

An alternative method that can detect both active and recovered patients is based on antibody detection. These technologies detect the immune system’s response to the infection.^56^ Therefore, the first step in developing the assay is antibody discovery^21,29,44,57,58^ and then establishing a reliable and high affinity antibody production line. In addition, while the terms “antibody” and “immunoglobulin” (Ig) are commonly used interchangeably, the immune system produces several types of antibodies depending on the type of immune response and pathway.^56^ If sufficient information is known about the temporal nature of the immune response, a diagnostic that indicates both the presence and the progression of a disease can be developed, improving the diagnostic. An example of this approach is detecting both the IgM and IgG antibodies that are produced at different timepoints during infection (Fig. 2).^56^ However, these antibodies have very different structures and affinities, which contribute to their precision and accuracy when used in a sensor. Therefore, it is important to understand their biochemistry before architecting a sensor that relies on this pair.

**FIG. 4.**
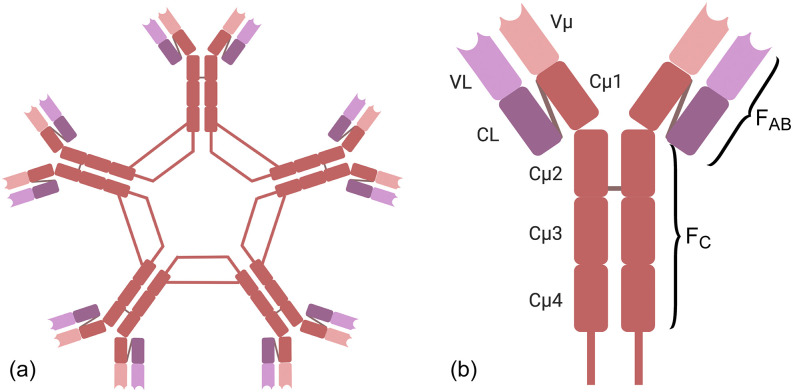
Immunoglobulin structure. (a) IgM structure is comprised of five monomers with ten binding sites. (b) IgG structure is comprised of one monomer with two binding sites. The binding sites are located at the end of the F_AB_ region of the monomer, in the variable region. Images created with Biorender.com.

IgM is the largest antibody (Fig. 4), comprised of five monomers arranged in a ring, resulting in ten binding sites. It is poly-reactive and has low-avidity, allowing it to respond quickly to unknown insults. The low avidity is fundamental to IgM’s operational principle and allows it to be the fastest responding antibody of the immune system. However, in the context of diagnostics, this property can result in high false positives. At the same time, the rapid production of these IgM’s allows them to be an indicator of active or recent infection, given their short lifetime. It takes the immune system ∼1 week to produce IgM’s, and they only remain in the circulatory system for approximately another week.

In contrast, IgG is comprised of a single monomer with two adaptive binding sites (Fig. 4). Because it is generated in response to a specific insult, it has moderate to high avidity for that insult. While it takes a few weeks for IgG antibody concentrations to rise and stabilize, IgG’s can circulate at a constant concentration for several months to a lifetime. Therefore, they provide a good indicator of past exposure. However, it is important to note that the concentration and avidity of IgG’s generated can be related to the magnitude of the initial insult and the host immune system response. Therefore, in the case of COVID-19, the concentration detected is not a reliable indicator of time since exposure because neither of these variables are known. On a side note, IgG’s are also responsible for the long-term immunity provided by engineered vaccines, which induce a strong immune system response. However, it is not clear at this time if the presence of COVID-19 IgG’s indicates long-term immunity.^59^

The majority of commercialized optical sensors for COVID-19 antibody detection have focused on leveraging existing instrumentation for signal readout, such as microplate readers or fluorescent imaging systems, or on developing point-of-care systems, including simple colorimetric indicators. These strategies vary in their approach for IgG and IgM identification, the complexity of the sample handling and chemistry protocols, the time-to-result, and the information that can be provided (Table I). All of these factors contribute to the false positive and false negative rates, which determine the accuracy and precision (or reliability) of the finding. Therefore, it is important to recognize that the term “antibody test” is a very broad classification given a large variety of diagnostic tests that rely on the detection of antibodies.

**TABLE I.** Overview of common diagnostic tests for COVID-19.

		Time for	Antibody	Concentration	Effectiveness
Test	Mechanism	the test	presence	of antibodies	of antibodies
Rapid diagnostic	Substrate changes color to indicate	10 min–30 min	Yes	No	No
test^44^	the presence of antibody				
Neutralization	Patient sample and virus are mixed	3 days–5 days	Yes	No	Yes
assay	with cells to determine the presence and				
test^60^	efficacy of protecting cells				
ELISA,^53,61^	Substrate changes or emits color a series	2 h–5 h	Yes	Yes	No
chemiluminescent	to indicate the presence of antibody;				
immunoassay	of dilutions are run to obtain concentration				

**FIG. 5.**
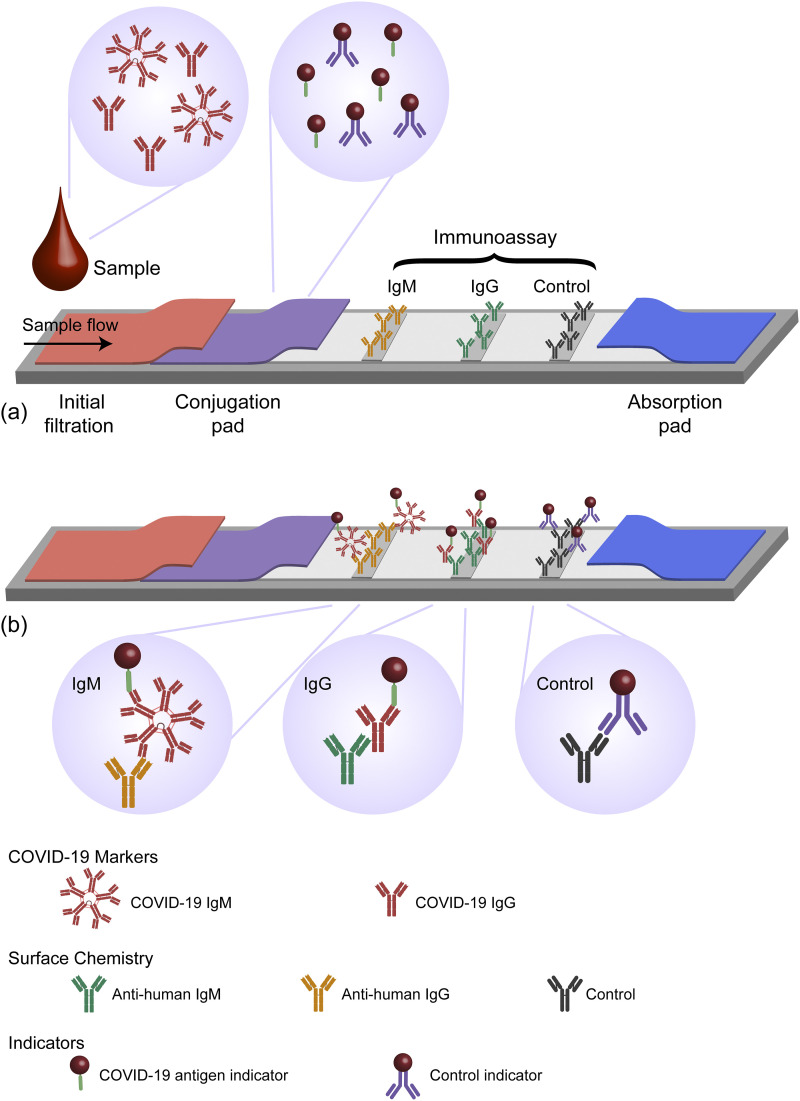
Schematic of the RDT. (a) The RDT has two diagnostic lanes and a control lane. The conjugation pad contains a COVID-19 antigen and a control antibody, both labeled with a metal nanoparticle. The sample is wicked across the conjugation pad and then across all three lanes. (b) If a strip changes color, it indicates that the antibody is present. Images created with Biorender.com.

Among the different antibody diagnostic methods, the rapid diagnostic test (RDT) is the most commonly recognized by the general public.^19,39,51,53^ An example configuration of an RDT is shown in Fig. 5. By simply monitoring the color of the two detection strips (as well as the control strip), the user can make a diagnosis in 10 min–30 min with a small sample of blood. Not surprisingly, due to their quick response time, low cost, and ease of use, these tests have quickly gained popularity. However, due to limitations with specificity of the reactants, the false positive and false negative rates are significantly higher than those in RT-qPCR.^14^ Therefore, while RDTs can be used as one piece of information, decisions regarding healthcare should not rely solely on antibody test results until higher affinity antibodies have been developed and can be reliably manufactured.^62^

In diagnosing and monitoring COVID-19 progression, a base set of physical symptoms that are simple to analyze has been identified, including temperature and blood oxygen saturation levels among others.^62^ While both measurements can indicate multiple other illnesses, they can still provide information quickly and inexpensively, without requiring blood samples, nasal swabs, or expensive equipment. Additionally, once diagnosed, the measurements can allow disease progression to be tracked from home, reducing the burden on the healthcare system.

Stand-off cameras based on infrared (8 *µ*m–14 *µ*m) detectors have been used extensively in the scientific community for some time to perform thermal imaging, particularly in the atmospheric and aerospace communities but also in environmental sciences for tracking global climate change. However, these detectors were extremely large and expensive. Several years ago, in response to SARS, stand-off imaging systems leveraging these thermal cameras were developed for airports and other high-traffic areas to easily and quickly monitor the temperatures of large populations of people. However, the shift to handheld units required a significant reduction in size and in cost as well as the integration of self-referencing capability. This combination was only recently accomplished. Currently, stand-off measurements for monitoring temperature are a fundamental component of many corporate and government COVID-19 monitoring strategies. However, body temperature is not a perfect indicator as asymptomatic carriers can transmit the virus yet have no discernable temperature increase.

Blood oxygen saturation monitoring, also known as pulse oximetry (or pulse-ox), is a way to determine the percent of hemoglobin that carries oxygen. This measure is an indicator of many physical parameters, including lung function. While the most accurate method is to directly perform a gas analysis of arterial blood, this approach is also incredibly invasive and rarely performed. The standard of care is to measure the peripheral oxygen saturation level. While systems based on monitoring changes in reflectance and in transmission have been developed, measuring transmission is more commonly used due to its higher accuracy. Specifically, the optical absorption of oxygenated hemoglobin is approximately an order of magnitude higher than that of de-oxygenated hemoglobin in the red blood cells (620 nm–700 nm), but in the near-IR, the values are nearly equal (800 nm–940 nm). Therefore, by comparing the two signals, the percent of oxygenated hemoglobin can be calculated.

The initial optical pulse-ox systems date back to the early 1930s and 1940s, but the systems were not in routine use in a medical setting until the 1970s and 1980s. Moreover, these systems relied on precision optical sources and were extremely sensitive to patient motion, limiting their use to hospital settings. In the 1990s, signal analysis technology was developed to stabilize the signal against patient motion. However, these systems still relied on precision light sources and complex control systems. With the advent of low-cost micro-LEDs with low power requirements that could be directly integrated on-chip, finger-clip pulse-ox devices were designed and developed. Inexpensive and suitable for at-home use, these systems have transformed cardiovascular care in both the hospital and the home. Given the impact of COVID-19 on lung function, these easy to use pulse-ox devices are now being used to monitor the progression of COVID-19 patients from home,^63–65^ and research is investigating their use as an “early warning system” for COVID-19.^66^

## III. EMERGING DIAGNOSTIC TECHNOLOGIES

A prominent area in integrated photonics focuses on the development of biological and chemical sensors for diagnostics for a wide range of diseases.^34,37,47,67–71^ Unlike the methods already discussed, the emerging technologies directly leverage light–matter interactions in the detection mechanism and have the possibility of being integrated with microfluidics for high-throughput sample delivery and analysis. High-throughput or multiplexing capability is of particular interest given diagnostic test shortages faced early on in the COVID-19 pandemic.^8^ Two commonly used methods to detect and identify specific substances in clinical samples are the detection of refraction index and optical transmission changes and the detection of optical scattering.

Beyond the well-known Surface Plasmon Resonance (SPR) biosensor originally commercialized by Biacore, new sensing systems based on plasmonic nanotechnology^72,73^ and silicon-based photonic rings and waveguides^74,75^ are continuously emerging to provide the most appealing analytical features for rapid screening and diagnosis. As nanofabrication methods and optical component integration have advanced, the portability of these platforms has improved. Both sensors rely on the fundamentally simple concept of evanescent field detection of refractive index change.

Briefly, an evanescent wave generated at the interface of a waveguide or a metallic nanostructure with the outer medium is able to probe minute variations of the dielectric refractive index and transduce them into variations of certain light properties, such as resonance, intensity, or phase. By tethering specific bioreceptors (e.g., antibodies or DNA probes) onto the sensor, the target analyte is captured from the sample. In this manner, the same surface chemistry methods developed for the commercialized RDTs can be leveraged to accelerate the design and translation of these optical systems. Once the target analyte attaches to the sensor surface, the refractive index of the local environment changes. Over a given concentration range, this change scales linearly with the analyte concentration. Because of the role that available surface binding sites play in generating the detection signal, one approach for controlling a sensor’s operating range is to alter the density and composition of binding sites. The idea of optimizing the surface chemistry to tailor the working range is an emerging area of research.

The evanescent wave optical detection schemes with the highest sensitivity are those that track the frequency shift of resonance or interferometry signals. Both signals are fundamentally monitoring the local change in the refractive index caused by an analyte. Resonance-shift methods monitor the change in a system’s resonance frequency, such as that of a plasmonic nanoparticle or waveguide, due to a local change in the refractive index caused by an analyte. Interferometric methods monitor the phase change of an optical probe signal due to local changes in the refractive index caused by an analyte.^35,37,49^ This approach delivers quantitative data in real time without the need of any fluorescent or colorimetric labeling (i.e., label-free assay). The increased sensitivity allows for a reduction in the requisite sample volume and reagents required, making them ideal tools for decentralized and high-throughput testing.^74,76^

In the last decade, label-free integrated photonic biosensors have demonstrated their capabilities in analyzing clinically relevant materials and reporting specific detection of proteins, nucleic acids, or pathogens in human body fluids (serum, urine, saliva, etc.) with outstanding assay sensitivity ranging from attomolar to femtomolar (aM–fM) levels of RNA molecules to less than ten bacterial cells, for example.^76–79^ They have also formed the foundation of portable diagnostic systems for viral pathogen detection for Ebola and malaria. This proven feasibility together with their unique versatility positions photonic biosensor technologies as an attractive solution for novel COVID-19 diagnostics.

Notably, optical technologies are being developed for two types of detection of a SARS-CoV-2 viral infection: (1) direct and (2) indirect. With direct detection, either the circulating viral RNA or the virus itself is detected. The first approach is similar to RT-qPCR in that the viral RNA is being detected. For these RNA sensors, specific RNA target sequences are identified by hybridization to a complementary nucleic acid sequence (i.e., DNA) immobilized on the surface. If the biosensor is sensitive enough, it does not need pre-amplification cycles based on PCR. This strategy represents a significant improvement over RT-qPCR and decreases the time-to-result from over an hour for RT-qPCR to 10 min–15 min (after RNA extraction). However, similar to qPCR, the sensor is detecting the copies of viral RNA per ml of blood. The second approach directly detects the circulating active viral particles, circumventing the basic limitation of PCR that has resulted in false positives. Namely, PCR amplifies both circulating RNA and RNA in active viral particles. Therefore, after a patient recovers, there can still be circulating RNA that can be amplified and detected. This circulating RNA can result in false positive signals. By detecting only intact viral particles, this limitation is overcome.

Previous research on photonic biosensors has demonstrated the genomic analysis of other respiratory virus infections: influenza, respiratory syncytial virus (RSV), and other coronaviruses such as the original SARS-CoV or the human coronavirus OC43/229E, responsible for common cold.^41,80^ However, in those cases, it was necessary to perform prior or post-amplification procedures to enhance the assay sensitivity. In general, genomic-based optical biosensing assays could provide an alternative method to RT-qPCR, meeting the sensitivity and specificity requirements for clinical diagnosis.

**FIG. 6.**
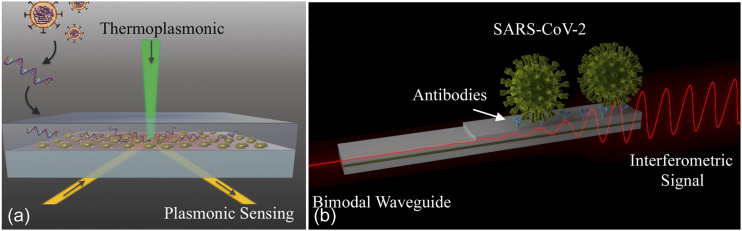
(a) Illustration of a dual-functional nanoplasmonic biosensor for COVID-19 RNA analysis. Reproduced with permission from Qiu *et al.*, ACS Nano **14**, 5268 (2020). Copyright 2020 American Chemical Society. (b) Illustration of a nanophotonic bimodal waveguide interferometer for direct sensing of intact SARS-CoV-2 viruses. CoNVaT project.

Soon after the COVID-19 outbreak, scientists started developing photonic biosensors for the detection of the SARS-CoV-2 genomic material.^81^ In the previous work, the general strategy of direct viral RNA detection using optical technologies has been demonstrated using plasmonic and silicon photonic biosensors. Notably, this approach allows for quantification of the viral load, and overall sensitivities in the range of 10^2^ copies/ml have been demonstrated.^82–84^ This prior work formed a foundation for developing detection technologies for SARS-CoV-2, and one of the first works published during the pandemic reported a nanoplasmonic biosensor for direct analysis of specific RNA fragments (RdRp gene) of the novel coronavirus.^81^ The sensor, based on gold nanoislands, combines the localized SPR sensing with the plasmonic photothermal effect that enhances the selective hybridization of complementary sequences while reducing non-specific binding of similar targets [Fig. 6(a)]. Qiu *et al.* showed a detectability in the picomolar (pM) range, estimating a limit of detection for entire viral RNA strands around 10^4^ copies/ml. The biosensor’s sensitivity could theoretically be appropriate for direct testing of clinical specimens without PCR given that the viral load in throat/nasal swabs of COVID-19 positive patients is usually between 10^5^ and 10^6^ copies/ml.

However, direct detection of the live virus particles is the ultimate goal. By functionalizing the sensor surface with specific receptors (e.g., antibodies) toward external proteins of the virus membrane, it is possible to capture intact virus entities that circulate in the body fluids, providing straightforward information of the live viral load in the patient without the need for RNA extraction and/or fragmentation procedures. To our knowledge, nothing has been reported yet for the direct detection of SARS-CoV-2 viruses with photonic biosensors, which is not surprising since the assay development requires high-quality, specific antibodies that are more difficult to produce than nucleic acid probes. However, several substantial research efforts are underway.

CoNVat, one of the first large research projects in Europe specifically dedicated to develop advanced nanophotonic biosensors for diagnosis of coronavirus infection, is focused on waveguide sensors. In particular, the goal of the research is to implement pioneering silicon photonics interferometric technology, the Bimodal Waveguide (BiMW) biosensor,^38^ for SARS-CoV-2 detection [Fig. 6(b)]. This technology will offer an integrated approach for accurate and quantitative diagnosis in less than 30 min.

One optical detection system has already proven effective in detecting viruses, including Ebola and Marburg. It is based on a simple interferometric reflectance imaging sensor (IRIS) platform.^13,85–91^ The IRIS can not only detect but also count individual viral pathogens in a complex medium without time-consuming sample purification or preparation. The IRIS has a unique detection mode from the previously discussed refractometric and fluorescence-based systems. Notably, when the virus binds to the surface, its weight generates a detectable optical signal. This is a label-free approach, and the detection signature is specific to the physical size and properties of the virus, providing a secondary signature for identification. In the previous work, the researchers demonstrated a real-time detection limit of 100 PFU/ml for vesicular stomatitis virus (VSV), and in response to the previous Ebola epidemic, they developed a 20 min assay for the Ebola virions at 1.5 × 10^4^ PFU/ml sensitivity corresponding to an average cycle threshold (CT) of 23.1 on RT-PCR.^90^ Therefore, once optimized for SARS-CoV-2 detection, this optical system is ideally suited to accelerate diagnosis.

In addition to direct detection of the viral load, optical sensors can also be used for indirect detection diagnostics or the detection of the body’s antibody response.^35,37,67,69^ As previously discussed, this type of assay is well-developed, and several relatively simple constructs that can provide a simple yes/no determination have been deployed. In contrast, photonic biosensors can quantify serum antibodies. In previous epidemics and pandemics, optical biosensors were developed for serological analysis of viral infections^45,92–94^ or have been used during vaccine development.^95–97^ In the current COVID-19 pandemic, the development of biosensing protocols for simple, rapid, and efficient serology testing could enable biomedical and epidemiology research efforts, accelerating the discovery of an optimum vaccine and improving our understanding of SARS-CoV-2 in humans.

However, like any emerging research technologies, there are numerous hurdles that must be overcome before these potentially transformative devices can be translated. Notably, the integration and full automation of operational sensing devices will require the establishment of universal and reproducible protocols for bioassay performance and the demonstration of reliability in large clinical studies. In some cases, multiple optical components, including the sensing elements as well as on-chip optical sources and detectors or imagers, may be needed. By creating a cohesive package, the noise level can be reduced, improving the overall system performance. This level of system complexity will leverage recent advances in heterogeneous fabrication protocols being developed. Additionally, many of these systems can also be integrated with complementary electrical components, such as electrophoresis, as well as microfluidics to move some aspects of sample preparation on-chip.

## IV. DISINFECTION TECHNOLOGIES

While this immediate crisis is the catalyst, there is a global need for the development of improved disinfection methods. Historically, the majority of antibiotic-resistant bacterial infections originated in medical settings. However, in 2019, there was a dramatic shift. Due to the heroic efforts of the medical community to improve disinfection and sterilization, the number of infections originating in medical settings decreased.^98,99^ However, the overall numbers continued their upward trajectory, due to community-based transmission. Moreover, many disinfection protocols assume ready access to chemicals and generate significant environmental waste. Therefore, to increase access to disinfection protocols in low-resource environments, reduce the environmental impact, and increase the usability, alternative methods must be developed and evaluated.^100–102^

**FIG. 7.**
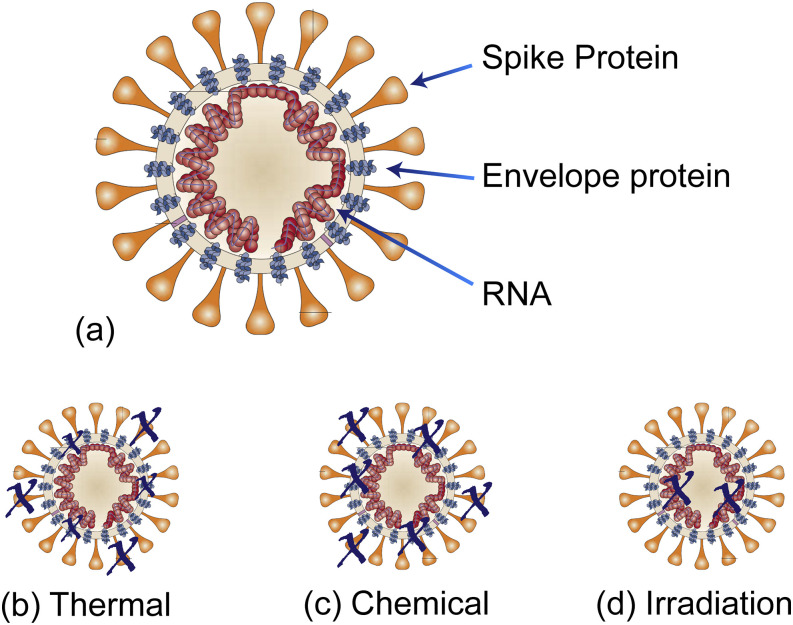
Overview of disinfection strategies. (a) Basic structure of coronavirus (and SARS-CoV-2), highlighting the key components needed for functioning. (b) Thermal and (c) chemical disinfection methods degrade the spike protein and the envelope protein. (d) Irradiation including UV-C degrades the RNA. Adapted with permission from Stadler *et al.*, Nat. Rev. Microbiol. **1**, 209 (2003). Copyright 2003 Springer Nature. SARS-beginning to understand a new virus.

When designing a disinfectant method, the first step is to evaluate the key components of the biological contaminant. In the case of coronavirus, the critical elements that ensure the stability, replication, and cell targeting ability are the envelope protein, RNA, and spike glycoprotein, respectively.^30,103–105^ If any of these components are destroyed, the virus will be inactivated. Therefore, most disinfection methods are designed to target one or more of these elements. There are three general categories: thermal, chemical, and radiation (Fig. 7). While the focus of this review is on radiation-based methods, for comparison purposes, all three methods will be briefly discussed.^102^

Thermal methods primarily target the spike glycoprotein with secondary impact on the envelope protein, depending on the thermal dose (heat and duration). Thermal methods were probably the first disinfection method developed and can be as simple as boiling water or an open flame. However, more rigorously controlled systems rely on ovens or other large chambers and can be either dry or moist heat. Given that they uniformly treat a sample, they are particularly suitable for linens, though they can be used for a myriad of supplies, such as surgical tools.

Chemical methods, including hydrogen peroxide and chlorine, target the spike glycoprotein and the envelope protein.^106,107^ Both vapor-based and handwipe chemical methods have been developed. Due to their efficacy in removing both monolayers and larger quantities of contaminants quickly, wipe-based chemical methods are the preferred disinfection methods for surfaces or solid materials. However, one drawback of chemical methods is that they only destabilize viruses that they directly interact with. To improve efficacy in materials with complex topologies, such as linens, a hybrid system is frequently used, combining both thermal and chemical methods.

While technically being a chemical method, due to its distinct mechanism, ozone (O_3_)-based disinfection is its own category. As a powerful oxidant, ozone is able to destroy the glycoproteins and the envelope proteins as well as all nucleotide bases comprising the RNA. Because ozone is a gas, it is effective on porous media as well as solid surfaces. Additionally, the process does not require high temperatures, making it particularly attractive. However, ozone is extremely reactive and readily returns to oxygen gas (O_2_). Therefore, it is very challenging to use.

The most recent development in disinfection systems is radiation-based systems. This category includes microwave, infrared (IR), and UV-C systems. Microwave-based and IR-based systems typically operate in an indirect manner. Namely, the microwaves (or radio-frequency waves) excite water, which thermally heats and destroys the spike glycoprotein. Thus, microwave radiation can be considered a moist thermal disinfection. Similarly, since IR sources are thermal sources, IR-systems can act as either dry or moist thermal systems. In contrast, systems based on UV-C radiation operate in a completely different manner.

RNA is comprised of four nucleotides: Adenine (A), Guanine (G), Uracil (U), and Cytosine (C). They are grouped into two categories: purines (A, G) and pyrimidines (U, C). Typically, purines bind to pyrimidines (A–U, G–C). However, when RNA is exposed to 260 nm light, it initiates binding between pyrimidines.^108,109^ By changing the fundamental structure of the RNA helix, RNA replication is inhibited, preventing viruses from reproducing. Therefore, similar to chemical methods, the UV-C light must directly interact with a virus particle to be effective. However, unlike chemical methods, UV-C does not expose the material to any kind of toxic chemical. Additionally, it can be performed at low temperatures. For these reasons, the initial application of UV-C was in the environmental field, namely, for air purification and water disinfection.^109–114^ However, the airborne contagiousness of SARS-CoV-2 increased the demand for new universal disinfection techniques to be developed.

In the context of COVID-19, UV-C has proven particularly effective in disinfecting PPE that protects healthcare workers against airborne pathogens, primarily masks, face shields, and eyewear. This ability has helped address the unprecedented spike in demand for those types of PPE that has threatened the safety of healthcare workers. While governments worked to relax import restrictions to ease the shock to PPE supply chains, during the worst part of the initial COVID-19 crisis, healthcare workers in many countries were forced to reuse their PPE at a dramatic scale. Although many countries are past the initial COVID-19 peak, outbreaks are still expected to occur throughout the world until a vaccine is widely available. This means that spurious PPE shortages will be a continuing reality for the time being, and it is worthwhile to investigate strategies that can enable safe PPE reuse in times of crisis. UV-C provides one path to disinfect PPE.

However, it is important to note that due to its universal damage mechanism that destroys both DNA and RNA, UV-C can have significant negative health effects on people as well as viruses, including permanent blindness.^108^ Therefore, there are numerous safety standards governing the use of UV-C light sources in the workplace. For example, automatic shut-offs and the use of eye-protection when using UV-C light sources are standard. However, many of these safety standards are missing in consumer products that do not undergo the same scrutiny. Given the rapid deployment of UV-C based technologies into the consumer market, the lack of safety standards is a growing concern.

One significant advantage of both thermal and radiation methods over chemical methods is that both thermal and radiation have minimal consumables. This difference is notable for several reasons. First, the amount of waste generated and the resulting environmental impact are decreased as compared to those in chemical techniques. Additionally, the reliance on supply chains and on manufacturing is reduced.

Unlike thermal treatments, UV disinfection protocols do not require large ovens, and the fundamental mechanism is amenable to high-throughput rates. They also may not necessarily require a large financial investment or lead time prior to their use because UV disinfectant tools come in a variety of forms, ranging from small handheld wands to semi-autonomous robotic systems. A field hospital established in 48 hours to deal with a COVID-19 outbreak may not be able to support the industrial-scale ovens with high power requirements for thermal disinfection or the chemical vapor stations with gas purification needs for chemical disinfection, but it could purchase portable UV disinfection wands and enclosures. Depending on the emitted UV power, the light simply needs to be held above the contaminated surface for a fixed amount of time to ensure that the surface has been sterilized. This can be as little as 10 s–30 s depending on the type of UV source used.^17,100,115–117^

UV-C sources fall into two common categories: bulbs and LEDs. Xenon or mercury bulbs are the most commonly used, having originally formed the foundation of the industry. While they are relatively inexpensive to make, they have high power requirements, which translate to high operating (or electrical) costs. Additionally, in comparison with that of alternative sources, their lifetime is short, and power cycling can further impact the lifetime. An emerging alternative is UV-C LEDs. High power LEDs have significantly reduced power demands and increased lifetime. They also have smaller footprints, allowing more compact (or handheld) systems to be designed. However, they are much more expensive, increasing the “upfront” costs as compared to bulbs. Last, despite being called “high power,” the LED output power is low to moderate when compared to that of bulbs. To account for this difference, the exposure time when using LEDs is increased in order to achieve the same dose.^117,118^

Dosage is the product of intensity delivered to a surface and time. Physical factors such as the distance between the source and the surface and the source intensity profile play a role in the calculation of how long a UV disinfectant tool needs to be used in order to be effective against different pathogens. Further complicating issues, different surface types (e.g., porous vs non-porous) require different dosages. Given the mechanism of UV-C, biological factors also contribute. Namely, because UV-C is initiating dimerization of the pyrimidines, if the concentration of pyrimidines is higher, a lower dose can be used. Thus, pathogens containing DNA, which is a double helix structure, intrinsically require a lower dose than RNA-based pathogens, including viruses. For this reason, viral pathogens require the highest dosages.

Last, there are two competing considerations: the desire to disinfect (damage) all viral and bacterial contaminants and the need to not damage the surface, particularly for repeated exposures. Therefore, determining the optimum dosage is a complex optimization problem that must consider not only the specific pathogen but also the UV-C source, the surface properties, and the specific application.

In the current climate, the two most common PPE disinfected using UV-C is N95 masks and face shields. These represent two very different surfaces, and their mechanism of acting as PPE is very different. Face shields are either glass or chemically resistant plastic, whereas N95 masks are fibrous (natural or manmade). Therefore, while the face shield surface is considered non-porous and robust against relatively high doses of UV-C (when compared to porous materials), N95 masks and surgical masks are more delicate. Additionally, while chemical treatments, including chemical immersion and wipes, can be used on face shields, chemical compatibility can impact the porous structure of N95 masks, degrading the filtration ability, particularly with repeated chemical disinfection. As a result, systems are typically configured for either masks or shields, but not both. Similarly, disinfection protocols are typically optimized for a single material type.^119^

Thus far, three general approaches for PPE disinfection have been studied: (1) reconfiguring standard biosafety cabinets,^16^ (2) creating enclosed portable boxes with a single UV-C source or arrays of UV-C sources,^17^ and (3) building large rooms with intense UV-C sources located in the center. The first two approaches are capable of disinfecting a few to a few dozen N95 masks or face shields at a time, while the third technique can disinfect hundreds of N95 masks at once. However, the infrastructure required for the third method is significantly more expensive.

Given the important role that the material plays in the N95 mask filtration efficacy, the UV-C dosage thresholds for material degradation have been extensively researched. In one study using biosafety cabinets as the UV-C source, the balance between the required dose to disinfect and the mask integrity was investigated. It was shown the sterilizer cabinet had a slight impact on the filtration efficiency of N95 masks (from the standard 95% to 93%) after 20 cycles. For an N95 mask, ∼4.5 J/cm^2^ is the lowest dosage at which no physical damage to N95 masks was observed, but this varies substantially and values up to 120 J/cm^2^ may be acceptable in certain situations.^15^ This value establishes a “damage threshold” rule of thumb for certain types of PPE.

Additionally, the environment around the mask during disinfection can play a role in damage. It is common to perform thermal treatments in a moist or humid environment as the humidity accelerates the disinfection process. However, the humidity also damages the masks, reducing the filtration to 80% after ten cycles.^15^ When this value is compared to 95% filtration for a dry oven or the UV sterilization cabinet, it is evident that the environment is a key factor that should be considered and controlled.

While the material damage threshold provides an upper limit to the total dose, the lower limit is set by the minimum dosage required to disinfect a surface. In one study, it was established that a 17 mW/cm^2^ treatment for 30 min (30.6 J/cm^2^) was enough to safely disinfect N95 masks against viral particles, allowing for ∼20 cycles of this treatment before physical damage occurred (establishing a damage threshold around ∼600 J/cm^2^).^15^ This work provides the current basis for all UV-based disinfection techniques when using N95 masks.

Given their reliability and environmental control, biosafety cabinets and fixed enclosures are the standard approach for performing UV-C disinfection in healthcare settings. However, UV-C is also particularly amenable to creating handheld platforms for consumer use. UV wands and small boxes for cellphones and other personal items are battery-powered and are ideal for resource constrained healthcare settings as well as personal use.

To disinfect larger surfaces around a room and objects that cannot be moved, a mobile system is needed. Therefore, it is desirable to create an autonomous or a semi-autonomous platform that can disinfect these rooms or other large spaces routinely. In general, current UV-C systems suitable for this task largely fall into one of the two categories: passive robots that operate simply within their environments and active robots that interact with components of their environments. Each type of robot has its own advantages and disadvantages associated with its development, implementation, and utilization.

The passive robots are much simpler systems, whose designs typically involve a mobile base with fixed vertical UV-C sources and are usually marketed as accomplishing “whole-room disinfection.” A few of these systems existed commercially prior to the COVID-19 pandemic, motivated by the need to reduce hospital acquired antibiotic-resistant bacterial infections.^117,120–122^ The fairly simplistic design of the most basic models allowed the manufacturing of these robots to be rapidly increased, and systems were beginning to be broadly distributed within a few weeks of the pandemic being formally acknowledged worldwide.

The design is primarily determined by the degree of autonomy, and the majority of systems rely on either pulsed xenon lamps^100,123–125^ or mercury gas bulbs.^125^ One study has suggested that the continuous dose of UV-C light provided by the mercury bulbs is more effective at reducing pathogen levels than that provided by pulsed xenon lamps.^125^ These lights are mounted upright on a mobile base, which ranges from a simple wheeled base that has to be manually moved around to a robotic base that allows for more autonomous use. Many of these systems have built-in safety measures, including motion-detecting sensors or lidar to detect people and automatically shut off the bulbs to prevent harmful exposure. Some also integrate sensory data and use software-based methods to enhance and optimize the delivery of UV-C light throughout the room, such as measuring reflected UV-C light to ensure that the lowest dose delivered throughout the room meets the minimum dosage required for disinfection.^101^

Studies of these passive robots have confirmed some of the theoretical advantages of UV-C disinfection. Through their “no touch” disinfection process, they remove the human error from the process of disinfection and allow for more frequent cleanings since a person is not required to be present during operation of the robot. However, these robots still suffer from limitations due to safety concerns; because they uniformly emit UV-C light throughout a room, the room must be completely empty of people to avoid potential harm. In addition, because UV-C light diminishes in intensity proportional to the distance squared, some hospitals have found the added inconvenience of positioning high-touch surfaces (such as mobile computer stands, hospitals beds, and tables) around the UV-C robot^125^ or moving the UV-C robot to several different positions within a room^123^ to be necessary.

In addition, the inability of these passive robots to directly interact with their environments due to limited sensing and a complete lack of effectors significantly limits their disinfection capabilities to only exposed surfaces. As a result, key surfaces within hospital rooms that may be hidden from these passive robots remain dirty. These could include occluded, high-frequency contact surfaces, such as the surface of a counter hidden under a box of gloves, the inside of an ADA door handle, or the back of a door. One study has suggested that even some exposed surfaces are still not disinfected sufficiently, implying that the robots may need to be “smarter” and interact more with the environment to provide more thorough disinfection.^123^ While these robots still suffer from some limitations, the importance of the more simplistic design should not be understated, as it had allowed these robots to be successfully commercialized and deployed in hospitals, rendering them immediately available during the COVID-19 pandemic.

More recently, there has been a push to develop robots with end effectors to actively manipulate their environments to allow for more comprehensive UV-C disinfection, particularly for high-trafficked surfaces. These interactions can allow access to surfaces that were occluded to the light provided by the passive robots. For instance, a robot with arms that allow it to grab objects could move a box of gloves to disinfect the surface of a counter underneath it or to pick up a keyboard and expose it to UV-C light from different angles. A robot with UV-C lights mounted to an arm, unlike the fixed UV-C lights of the passive robots, could actively position its arm to disinfect surfaces that are difficult to access on immobilized objects or between objects.

Because these robots require more complex control algorithms to include the manipulation of their arms, development of these active robots has taken longer than the design of the passive robots, as evidenced by the availability of only passive robots in the commercial market. Furthermore, these robots are most advantageous if they are semi-autonomous or fully autonomous, freeing up a crucial staff member who would otherwise be occupied controlling or directing the robot. This requires the development of intricate planning algorithms, the integration of sensory input for the movement of these robots’ manipulators, and the design and construction of the system.

Since the COVID-19 pandemic began, one Swiss startup has been circumnavigating the long development period by adapting a previous design. Rovenso has existing designs for mobile robots for autonomous security and monitoring purposes, including a custom mobile base capable of navigating varying terrain and even climbing stairs. Using a self-described “hack,” the Rovenso team has attached a UV-C light source to their ROVéo robot, which was originally designed to autonomously monitor the security of industrial sites.^126^ Their design uses lidar to map the surrounding environments and target highly used surfaces for UV-C disinfection. However, Rovenso has designed their UV-C disinfection robot primarily for use in non-medical spaces where less stringent cleaning requirements are needed, such as offices and indoor workspaces.

Although UV-C cannot and should not replace thermal and chemical disinfection in every scenario, UV-C can be used effectively in many situations where thermal and chemical methods are not possible or practical. Additionally, given the low consumable requirements, UV-C is compatible with a wide range of operating environments and can provide an alternative method, particularly when supply chains are strained.

## V. FUTURE OUTLOOK AND CONCLUSION

Optical technologies have directly contributed to both preventing the spread of and diagnosing COVID-19. This is truly indicative of the ubiquitous nature of photonics technologies. While many of the technologies discussed here are already in the community and in healthcare settings, many are still in nascent stages of development with their impact yet to be realized. Two areas that will experience significant growth are the coupling between photonics and robotics for automated disinfection and photonics, robotics, and artificial intelligence (AI) for high-throughput diagnostics. Both robotics and AI are experiencing intellectual revolutions with synergistic advances, such as the development of autonomous robots. By combining these intelligent automated systems with photonic-based disinfection or diagnostic technologies, our approach to disinfection and diagnostics could be revolutionized.

As mentioned previously, one of the initial uses of UV-C disinfection was in environmental applications, including air and water purification, and this market remains the highest sector. Historically, the majority of these units were used in conjunction with filtration systems to break down and remove contaminants related to the spread of airborne mold or other asthma-related particulates. However, given that COVID-19 spreads via airborne transmission, the integration of UV-C into ventilation systems is one potential approach under consideration for reducing transmission in indoor settings. UV-C would be particularly attractive for this application given its ability to disinfect without direct manual intervention. However, several challenges for long-term, effective operation must be considered, including the total dose required, the cost of operation, and the ability to replace the UV-C source. In this type of system, high power UV LEDs would be the ideal source, given their low cost of operation, their stable emission intensity over the lifetime, and their long operating lifetime. However, the initial cost of high power UV LEDs is significantly more than that of mercury bulbs. Thus, advances in photonics manufacturing will greatly drive decision-making in this application. Last, it is important to note that this type of system would not prevent direct person-to-person transmission. In this context, the threat of infection from transmission through ventilation systems has not been rigorously established.

In a related application, performing disinfection of surfaces that we directly contact is manually intensive. As a result, it is challenging to maintain a clean environment in high-traffic areas, such as public transit, public bathrooms, and sports arenas. Therefore, these high contact points carry a high risk of transmission. An autonomous robot capable of operating independently would be able to reduce transmission risk by disinfecting more frequently. This ability would not only reduce infections during the present COVID-19 pandemic but also improve overall healthcare in the future, as many seasonal flu viruses are transmitted on surfaces. However, while this technology could have a clear positive societal impact, it is also important to recognize and address the safety and ethical concerns.^127,128^ For example, an autonomous robotic system that could potentially interact with people and pets would need to have integrated safety measures to ensure that they were not exposed to the UV-C source. In addition, discussions on the complex ethical landscape in AI and robotics are currently ongoing.

Throughout the COVID-19 pandemic, it has become evident that diagnosis is very complex, relying on advances in biotechnology as well as sensing methods. Additionally, there are many types of samples that can be analyzed. For example, while nasal swabs were the primary initial samples, more recent antibody tests rely on blood. This diversity motivated the development of a range of robotic sample handling systems that required smaller sample volumes and used less reagents per test. Additionally, the robotic systems accelerated the sample processing, enabling a significant increase in throughput. However, many of these robotic systems relied on proprietary plasticware and biological reagents. As a result, shortages in either supply impacted diagnostic labs and have motivated many facilities to develop their own supply chains. This dependence on outside vendors, particularly for key reagents, is always an important consideration when developing a new diagnostic.

As discussed, in the antibody tests, both false positives and false negatives can occur. Therefore, to improve accuracy, it is necessary to evaluate not just a single test result, but the result in the context of the patient’s other symptoms. Moreover, given the high transmission risk, the time-to-result, or the speed with which a diagnostic test produces a result, is paramount. While optical sensors produce results quickly, contextualization of the results relies on additional information. By combining these results with a machine learning-based AI optimized to contextualize the results, physicians will be armed with the tools needed to make higher accuracy treatment decisions on a faster timescale. Such a system not only would advance the present COVID-19 treatment strategy but also could change our approach to patient care.^62,129^ However, to be effective, machine learning systems require extensive learning libraries as well as a better understanding of the disease pathophysiology.^19,27,33^ Because COVID-19 is so new, obtaining this key information is extremely challenging.

## AUTHORS’ CONTRIBUTIONS

All authors contributed equally to this work.

## DATA AVAILABILITY

Data sharing is not applicable to this article as no new data were created or analyzed in this study.

## References

[1] J. de Anda, E. Y. Lee, C. K. Lee, R. R. Bennett, X. Ji, S. Soltani, M. C. Harrison, A. E. Baker, Y. Luo, T. Chou, G. A. O’Toole, A. M. Armani, R. Golestanian, and G. C. L. Wong, ACS Nano 11, 9340 (2017). 10.1021/acsnano.7b04738 28836761 PMC5978429

[2] F. Wang, H. Wan, Z. Ma, Y. Zhong, Q. Sun, Y. Tian, L. Qu, H. Du, M. Zhang, L. Li, H. Ma, J. Luo, Y. Liang, W. J. Li, G. Hong, L. Liu, and H. Dai, Nat. Methods 16, 545 (2019). 10.1038/s41592-019-0398-7 31086342 PMC6579541

[3] W. Zipfel, R. Williams, and W. Webb, Nat. Biotechnol. 21, 1368 (2003). 10.1038/nbt899 14595365

[4] D. M. Lukin, C. Dory, M. A. Guidry, K. Y. Yang, S. D. Mishra, R. Trivedi, M. Radulaski, S. Sun, D. Vercruysse, G. H. Ahn, and J. Vučković, Nat. Photonics 14, 330 (2020). 10.1038/s41566-019-0556-6

[5] A. Kovach, D. Chen, J. He, H. Choi, A. H. Dogan, M. Ghasemkhani, H. Taheri, and A. M. Armani, Adv. Opt. Photonics 12, 135 (2020). 10.1364/aop.376924

[6] A. N. Willner, P. Liao, K. Zou, Y. Cao, A. Kordts, M. Karpov, M. H. P. Pfeiffer, A. Almaiman, A. Fallahpour, F. Alishahi, K. Manukyan, M. Tur, T. J. Kippenberg, and A. E. Willner, Opt. Lett. 43, 5563 (2018). 10.1364/ol.43.005563 30439896

[7] R. Verity, L. C. Okell, I. Dorigatti, P. Winskill, C. Whittaker, N. Imai, G. Cuomo-Dannenburg, H. Thompson, P. G. T. Walker, H. Fu, A. Dighe, J. T. Griffin, M. Baguelin, S. Bhatia, A. Boonyasiri, A. Cori, Z. Cucunubá, R. FitzJohn, K. Gaythorpe, W. Green, A. Hamlet, W. Hinsley, D. Laydon, G. Nedjati-Gilani, S. Riley, S. van Elsland, E. Volz, H. Wang, Y. Wang, X. Xi, C. A. Donnelly, A. C. Ghani, and N. M. Ferguson, Lancet Infect. Dis. 20, 669 (2020). 10.1016/s1473-3099(20)30243-7 32240634 PMC7158570

[8] M. Satyanarayana, Chem. Eng. News (2020); available at https://cen.acs.org/analytical-chemistry/diagnostics/Shortage-RNA-extraction-kits-hampers/98/web/2020/03.

[9] Y. J. Kim, H. Sung, C.-S. Ki, and M. Hur, Ann. Lab. Med. 40, 349 (2020). 10.3343/alm.2020.40.5.349 32237287 PMC7169622

[10] A. S. Fauci, H. C. Lane, and R. R. Redfield, N. Engl. J. Med. 382, 1268 (2020). 10.1056/nejme2002387 32109011 PMC7121221

[11] C. del Rio and P. N. Malani, JAMA, J. Am. Med. Assoc. 323, 1339 (2020). 10.1001/jama.2020.3072 32108857

[12] D. Cereda, M. Tirani, F. Rovida, V. Demicheli, M. Ajelli, P. Poletti, F. Trentini, G. Guzzetta, V. Marziano, A. Barone, M. Magoni, S. Deandrea, G. Diurno, M. Lombardo, M. Faccini, A. Pan, R. Bruno, E. Pariani, G. Grasselli, A. Piatti, M. Gramegna, F. Baldanti, A. Melegaro, and S. Merler, arXiv:2003.09320 [q-Bio] (2020).

[13] A. M. Armani, D. E. Hurt, D. Hwang, M. C. McCarthy, and A. Scholtz, Nat. Rev. Mater. 5, 403 (2020). 10.1038/s41578-020-0205-1 PMC7212509 32395258

[14] I. Santiago, ChemBioChem (published online 2020). 10.1002/cbic.202000250

[15] L. Liao, W. Xiao, M. Zhao, X. Yu, H. Wang, Q. Wang, S. Chu, and Y. Cui, ACS Nano 14, 6348 (2020). 10.1021/acsnano.0c03597 32368894

[16] K. J. Card, D. Crozier, A. Dhawan, M. Dinh, E. Dolson, N. Farrokhian, V. Gopalakrishnan, E. Ho, E. S. King, N. Krishnan, G. Kuzmin, J. Maltas, J. Pelesko, J. A. Scarborough, J. G. Scott, G. Sedor, and D. T. Weaver, medRxiv:2020.03.25.20043489 (2020).

[17] R. C. She, D. Chen, P. Pak, D. K. Armani, A. Schubert, and A. M. Armani, Biomed. Opt. Express 11, 4326 (2020). 10.1364/boe.395659 32923046 PMC7449718

[18] L. Zou, F. Ruan, M. Huang, L. Liang, H. Huang, Z. Hong, J. Yu, M. Kang, Y. Song, J. Xia, Q. Guo, T. Song, J. He, H.-L. Yen, M. Peiris, and J. Wu, N. Engl. J. Med. 382, 1177 (2020). 10.1056/nejmc2001737 32074444 PMC7121626

[19] C. Y.-P. Lee, R. T. P. Lin, L. Renia, and L. F. P. Ng, Front. Immunol. 11, 879 (2020). 10.3389/fimmu.2020.00879 32391022 PMC7194125

[20] C. Wang, W. Li, D. Drabek, N. M. A. Okba, R. van Haperen, A. D. M. E. Osterhaus, F. J. M. van Kuppeveld, B. L. Haagmans, F. Grosveld, and B.-J. Bosch, Nat. Commun. 11, 2251 (2020). 10.1038/s41467-020-16256-y 32366817 PMC7198537

[21] Q.-X. Long, B.-Z. Liu, H.-J. Deng, G.-C. Wu, K. Deng, Y.-K. Chen, P. Liao, J.-F. Qiu, Y. Lin, X.-F. Cai, D.-Q. Wang, Y. Hu, J.-H. Ren, N. Tang, Y.-Y. Xu, L.-H. Yu, Z. Mo, F. Gong, X.-L. Zhang, W.-G. Tian, L. Hu, X.-X. Zhang, J.-L. Xiang, H.-X. Du, H.-W. Liu, C.-H. Lang, X.-H. Luo, S.-B. Wu, X.-P. Cui, Z. Zhou, M.-M. Zhu, J. Wang, C.-J. Xue, X.-F. Li, L. Wang, Z.-J. Li, K. Wang, C.-C. Niu, Q.-J. Yang, X.-J. Tang, Y. Zhang, X.-M. Liu, J.-J. Li, D.-C. Zhang, F. Zhang, P. Liu, J. Yuan, Q. Li, J.-L. Hu, J. Chen, and A.-L. Huang, Nat. Med. 26, 845 (2020). 10.1038/s41591-020-0897-1 32350462

[22] C. Liu, Q. Zhou, Y. Li, L. V. Garner, S. P. Watkins, L. J. Carter, J. Smoot, A. C. Gregg, A. D. Daniels, S. Jervey, and D. Albaiu, ACS Cent. Sci. 6, 315 (2020). 10.1021/acscentsci.0c00272 32226821 PMC10467574

[23] G. Lippi and M. Plebani, Clin. Chem. Lab. Med. 58, 1063 (2020). 10.1515/cclm-2020-0240 32191623

[24] S. Jiang, C. Hillyer, and L. Du, Trends Immunol. 41, 355 (2020). 10.1016/j.it.2020.03.007 32362491 PMC7271084

[25] J. Alijotas-Reig, E. Esteve-Valverde, C. Belizna, A. Selva-OʾCallaghan, J. Pardos-Gea, A. Quintana, A. Mekinian, A. Anunciacion-Llunell, and F. Miró-Mur, Autoimmun. Rev. 19, 102569 (2020). 10.1016/j.autrev.2020.102569 32376394 PMC7252146

[26] S. F. Ahmed, A. A. Quadeer, and M. R. McKay, Viruses 12, 254 (2020). 10.3390/v12030254 PMC7150947 32106567

[27] B. E. Young, S. W. X. Ong, S. Kalimuddin, J. G. Low, S. Y. Tan, J. Loh, O.-T. Ng, K. Marimuthu, L. W. Ang, T. M. Mak, S. K. Lau, D. E. Anderson, K. S. Chan, T. Y. Tan, T. Y. Ng, L. Cui, Z. Said, L. Kurupatham, M. I.-C. Chen, M. Chan, S. Vasoo, L.-F. Wang, B. H. Tan, R. T. P. Lin, V. J. M. Lee, Y.-S. Leo, D. C. Lye, and Singapore 2019 Novel Coronavirus Outbreak Research Team, JAMA, J. Am. Med. Assoc. 323, 1488 (2020). 10.1001/jama.2020.3204

[28] X. Tang, C. Wu, X. Li, Y. Song, X. Yao, X. Wu, Y. Duan, H. Zhang, Y. Wang, Z. Qian, J. Cui, and J. Lu, Natl. Sci. Rev. 7, 1012 (2020). 10.1093/nsr/nwaa036 PMC7107875 34676127

[29] S. Sun, X. Cai, H. Wang, G. He, Y. Lin, B. Lu, C. Chen, Y. Pan, and X. Hu, Clin. Chim. Acta 507, 174 (2020). 10.1016/j.cca.2020.04.024 32339487 PMC7194694

[30] J. Shang, Y. Wan, C. Luo, G. Ye, Q. Geng, A. Auerbach, and F. Li, Proc. Natl. Acad. Sci. U. S. A. 117, 11727 (2020). 10.1073/pnas.2003138117 32376634 PMC7260975

[31] G. Li and E. De Clercq, Nat. Rev. Drug Discovery 19, 149 (2020). 10.1038/d41573-020-00016-0 32127666

[32] T. T.-Y. Lam, N. Jia, Y.-W. Zhang, M. H.-H. Shum, J.-F. Jiang, H.-C. Zhu, Y.-G. Tong, Y.-X. Shi, X.-B. Ni, Y.-S. Liao, W.-J. Li, B.-G. Jiang, W. Wei, T.-T. Yuan, K. Zheng, X.-M. Cui, J. Li, G.-Q. Pei, X. Qiang, W. Y.-M. Cheung, L.-F. Li, F.-F. Sun, S. Qin, J.-C. Huang, G. M. Leung, E. C. Holmes, Y.-L. Hu, Y. Guan, and W.-C. Cao, Nature 583, 282 (2020). 10.1038/s41586-020-2169-0 32218527

[33] O. Altay, E. Mohammadi, S. Lam, H. Turkez, J. Boren, J. Nielsen, M. Uhlen, and A. Mardinoglu, iScience 23, 101303 (2020). 10.1016/j.isci.2020.101303 32622261 PMC7305759

[34] S. E. McBirney, D. Chen, A. Scholtz, H. Ameri, and A. M. Armani, ACS Sens. 3, 1264 (2018). 10.1021/acssensors.8b00269 29781606

[35] S. Frustaci and F. Vollmer, Curr. Opin. Chem. Biol. 51, 66 (2019). 10.1016/j.cbpa.2019.05.003 31202140

[36] A. Raj and A. K. Sen, in Environmental, Chemical and Medical Sensors, edited by Bhattacharya S., Agarwal A. K., Chanda N., Pandey A., and Sen A. K. (Springer, 2018), pp. 389–408.

[37] S. Mehrabani, A. Maker, and A. Armani, Sensors 14, 5890 (2014). 10.3390/s140405890 24675757 PMC4029679

[38] D. Duval, A. B. González-Guerrero, S. Dante, J. Osmond, R. Monge, L. J. Fernández, K. E. Zinoviev, C. Domínguez, and L. M. Lechuga, Lab Chip 12, 1987 (2012). 10.1039/c2lc40054e 22538502

[39] K. K.-W. To, O. T.-Y. Tsang, W.-S. Leung, A. R. Tam, T.-C. Wu, D. C. Lung, C. C.-Y. Yip, J.-P. Cai, J. M.-C. Chan, T. S.-H. Chik, D. P.-L. Lau, C. Y.-C. Choi, L.-L. Chen, W.-M. Chan, K.-H. Chan, J. D. Ip, A. C.-K. Ng, R. W.-S. Poon, C.-T. Luo, V. C.-C. Cheng, J. F.-W. Chan, I. F.-N. Hung, Z. Chen, H. Chen, and K.-Y. Yuen, Lancet Infect. Dis. 20, 565 (2020). 10.1016/s1473-3099(20)30196-1 32213337 PMC7158907

[40] Y.-F. Chang, W.-H. Wang, Y.-W. Hong, R.-Y. Yuan, K.-H. Chen, Y.-W. Huang, P.-L. Lu, Y.-H. Chen, Y.-M. A. Chen, L.-C. Su, and S.-F. Wang, Anal. Chem. 90, 1861 (2018). 10.1021/acs.analchem.7b03934 29327590

[41] B. Koo, C. E. Jin, T. Y. Lee, J. H. Lee, M. K. Park, H. Sung, S. Y. Park, H. J. Lee, S. M. Kim, J. Y. Kim, S.-H. Kim, and Y. Shin, Biosens. Bioelectron. 90, 187 (2017). 10.1016/j.bios.2016.11.051 27894035 PMC7127409

[42] H. D. VanGuilder, K. E. Vrana, and W. M. Freeman, Biotechniques 44, 619 (2008). 10.2144/000112776 18474036

[43] T. Nolan, R. E. Hands, and S. A. Bustin, Nat. Protoc. 1, 1559 (2006). 10.1038/nprot.2006.236 17406449

[44] Z. Li, Y. Yi, X. Luo, N. Xiong, Y. Liu, S. Li, R. Sun, Y. Wang, B. Hu, W. Chen, Y. Zhang, J. Wang, B. Huang, Y. Lin, J. Yang, W. Cai, X. Wang, J. Cheng, Z. Chen, K. Sun, W. Pan, Z. Zhan, L. Chen, and F. Ye, J. Med. Virol. 92(9), 1518 (2020 10.1002/jmv.25727 PMC7228300 32104917

[45] H. Zhang and B. L. Miller, Biosens. Bioelectron. 141, 111476 (2019). 10.1016/j.bios.2019.111476 31272058 PMC6717022

[46] H. K. Hunt and A. M. Armani, IEEE J. Sel. Top. Quantum Electron. 20, 121 (2014). 10.1109/jstqe.2013.2272916

[47] A. L. Washburn, L. C. Gunn, and R. C. Bailey, Anal. Chem. 81, 9499 (2009). 10.1021/ac902006p 19848413 PMC2783283

[48] M. Shen, Y. Zhou, J. Ye, A. A. Abdullah AL-maskri, Y. Kang, S. Zeng, and S. Cai, J. Pharm. Anal. 10, 97 (2020). 10.1016/j.jpha.2020.02.010 32292623 PMC7102540

[49] H. K. Hunt and A. M. Armani, Nanoscale 2, 1544 (2010). 10.1039/c0nr00201a 20820687

[50] R. Weissleder, H. Lee, J. Ko, and M. J. Pittet, Sci. Transl. Med. 12, eabc1931 (2020). 10.1126/scitranslmed.abc1931 32493791

[51] S. K. Vashist, Diagnostics 10, 202 (2020). 10.3390/diagnostics10040202 PMC7235801 32260471

[52] D. Ferrari, A. Motta, M. Strollo, G. Banfi, and M. Locatelli, Clin. Chem. Lab. Med. 58, 1095 (2020). 10.1515/cclm-2020-0398 32301746

[53] L. J. Carter, L. V. Garner, J. W. Smoot, Y. Li, Q. Zhou, C. J. Saveson, J. M. Sasso, A. C. Gregg, D. J. Soares, T. R. Beskid, S. R. Jervey, and C. Liu, ACS Cent. Sci. 6, 591 (2020). 10.1021/acscentsci.0c00501 32382657 PMC7197457

[54] S. K. Yong, P. C. Su, and Y. S. Yang, Biotechnol. J. 15, 2000152 (2020). 10.1002/biot.202000152 PMC7267081 32419272

[55] T. Ishige, S. Murata, T. Taniguchi, A. Miyabe, K. Kitamura, K. Kawasaki, M. Nishimura, H. Igari, and K. Matsushita, Clin. Chim. Acta 507, 139 (2020). 10.1016/j.cca.2020.04.023 32335089 PMC7179514

[56] H. W. Schroeder, Jr. and L. Cavacini, J. Allergy Clin. Immunol. 125, S41 (2010). 10.1016/j.jaci.2009.09.046 20176268 PMC3670108

[57] M. Yuan, N. C. Wu, X. Zhu, C.-C. D. Lee, R. T. Y. So, H. Lv, C. K. P. Mok, and I. A. Wilson, Science 368, 630 (2020). 10.1126/science.abb7269 32245784 PMC7164391

[58] Y. Zhang, M. Xiao, S. Zhang, P. Xia, W. Cao, W. Jiang, H. Chen, X. Ding, H. Zhao, H. Zhang, C. Wang, J. Zhao, X. Sun, R. Tian, W. Wu, D. Wu, J. Ma, Y. Chen, D. Zhang, J. Xie, X. Yan, X. Zhou, Z. Liu, J. Wang, B. Du, Y. Qin, P. Gao, X. Qin, Y. Xu, W. Zhang, T. Li, F. Zhang, Y. Zhao, Y. Li, and S. Zhang, N. Engl. J. Med. 382, e38 (2020). 10.1056/nejmc2007575 32268022 PMC7161262

[59] N. Vabret, G. J. Britton, C. Gruber, S. Hegde, J. Kim, M. Kuksin, R. Levantovsky, L. Malle, A. Moreira, M. D. Park, L. Pia, E. Risson, M. Saffern, B. Salomé, M. Esai Selvan, M. P. Spindler, J. Tan, V. van der Heide, J. K. Gregory, K. Alexandropoulos, N. Bhardwaj, B. D. Brown, B. Greenbaum, Z. H. Gümüş, D. Homann, A. Horowitz, A. O. Kamphorst, M. A. Curotto de Lafaille, S. Mehandru, M. Merad, R. M. Samstein, M. Agrawal, M. Aleynick, M. Belabed, M. Brown, M. Casanova-Acebes, J. Catalan, M. Centa, A. Charap, A. Chan, S. T. Chen, J. Chung, C. C. Bozkus, E. Cody, F. Cossarini, E. Dalla, N. Fernandez, J. Grout, D. F. Ruan, P. Hamon, E. Humblin, D. Jha, J. Kodysh, A. Leader, M. Lin, K. Lindblad, D. Lozano-Ojalvo, G. Lubitz, A. Magen, Z. Mahmood, G. Martinez-Delgado, J. Mateus-Tique, E. Meritt, C. Moon, J. Noel, T. O’Donnell, M. Ota, T. Plitt, V. Pothula, J. Redes, I. Reyes Torres, M. Roberto, A. R. Sanchez-Paulete, J. Shang, A. S. Schanoski, M. Suprun, M. Tran, N. Vaninov, C. M. Wilk, J. Aguirre-Ghiso, D. Bogunovic, J. Cho, J. Faith, E. Grasset, P. Heeger, E. Kenigsberg, F. Krammer, and U. Laserson, Immunity 52, 910 (2020). 10.1016/j.immuni.2020.05.002 32505227 PMC7200337

[60] J. Nie, Q. Li, J. Wu, C. Zhao, H. Hao, H. Liu, L. Zhang, L. Nie, H. Qin, M. Wang, Q. Lu, X. Li, Q. Sun, J. Liu, C. Fan, W. Huang, M. Xu, and Y. Wang, Emerging Microbes Infect. 9, 680 (2020). 10.1080/22221751.2020.1743767 PMC7144318 32207377

[61] A. Padoan, C. Cosma, L. Sciacovelli, D. Faggian, and M. Plebani, Clin. Chem. Lab. Med. 58, 1081 (2020). 10.1515/cclm-2020-0443 32301749

[62] L. Wynants, B. Van Calster, G. S. Collins, R. D. Riley, G. Heinze, E. Schuit, M. M. J. Bonten, J. A. A. Damen, T. P. A. Debray, M. D. Vos, P. Dhiman, M. C. Haller, M. O. Harhay, L. Henckaerts, N. Kreuzberger, A. Lohmann, K. Luijken, J. Ma, C. L. A. Navarro, J. B. Reitsma, J. C. Sergeant, C. Shi, N. Skoetz, L. J. M. Smits, K. I. E. Snell, M. Sperrin, R. Spijker, E. W. Steyerberg, T. Takada, S. M. J. van Kuijk, F. S. van Royen, C. Wallisch, L. Hooft, K. G. M. Moons, and M. van Smeden, BMJ 369, m1328 (2020). 10.1136/bmj.m1328 32265220 PMC7222643

[63] R. G. Wilkerson, J. D. Adler, N. G. Shah, and R. Brown, Am. J. Emerging Med. (published online 2020). 10.1016/j.ajem.2020.05.044

[64] F. Michard, K. Shelley, and E. L’Her, J. Clin. Monit. Comput. (published online 2020). 10.1007/s10877-020-00550-7

[65] A. M. Luks and E. R. Swenson, Ann. Am. Thorac. Soc. 17, 918 (2020). 10.1513/annalsats.202004-327cme 32735170 PMC7393782

[66] R. Jouffroy, D. Jost, and B. Prunet, Crit. Care 24, 313 (2020). 10.1186/s13054-020-03036-9 32513249 PMC7278215

[67] Y. Zhang, T. Zhou, B. Han, A. Zhang, and Y. Zhao, Nanoscale 10, 13832 (2018). 10.1039/c8nr03709d 30020301

[68] M. E. Lee and A. M. Armani, ACS Sens. 1, 1251 (2016). 10.1021/acssensors.6b00491

[69] R. M. Hawk and A. M. Armani, Biosens. Bioelectron. 65, 198 (2015). 10.1016/j.bios.2014.10.041 25461158 PMC4408228

[70] S. E. McBirney, K. Trinh, A. Wong-Beringer, and A. M. Armani, Biomed. Opt. Express 7, 4034 (2016). 10.1364/boe.7.004034 27867713 PMC5102515

[71] D. Sevenler, O. Avci, and M. S. Ünlü, Biomed. Opt. Express 8, 2976 (2017). 10.1364/boe.8.002976 28663920 PMC5480443

[72] X. Han, K. Liu, and C. Sun, Materials 12, 1411 (2019). 10.3390/ma12091411 PMC6539671 31052240

[73] E. Mauriz, P. Dey, and L. M. Lechuga, Analyst 144, 7105 (2019). 10.1039/c9an00701f 31663527

[74] A. Fernández Gavela, D. Grajales García, J. Ramirez, and L. Lechuga, Sensors 16, 285 (2016). 10.3390/s16030285 26927105 PMC4813860

[75] C. Ciminelli, F. Dell’Olio, D. Conteduca, and M. N. Armenise, IET Optoelectron. 13, 48 (2019). 10.1049/iet-opt.2018.5082

[76] M. Soler, C. S. Huertas, and L. M. Lechuga, Expert Rev. Mol. Diagn. 19, 71 (2019). 10.1080/14737159.2019.1554435 30513011

[77] C. S. Huertas, O. Calvo-Lozano, A. Mitchell, and L. M. Lechuga, Front. Chem. 7, 724 (2019). 10.3389/fchem.2019.00724 31709240 PMC6823211

[78] J. Maldonado, M.-C. Estévez, A. Fernández-Gavela, J. J. González-López, A. B. González-Guerrero, and L. M. Lechuga, Analyst 145, 497 (2020). 10.1039/c9an01485c 31750459

[79] E. Luan, H. Shoman, D. Ratner, K. Cheung, and L. Chrostowski, Sensors 18, 3519 (2018). 10.3390/s18103519 PMC6210424 30340405

[80] L. Shi, Q. Sun, J. He, H. Xu, C. Liu, C. Zhao, Y. Xu, C. Wu, J. Xiang, D. Gu, J. Long, and H. Lan, Bio-Med. Mater. Eng. 26, S2207 (2015). 10.3233/bme-151448 26406000

[81] G. Qiu, Z. Gai, Y. Tao, J. Schmitt, G. A. Kullak-Ublick, and J. Wang, ACS Nano. 14, 5268 (2020). 10.1021/acsnano.0c02439 32281785

[82] B. A. Prabowo, R. Y. L. Wang, M. K. Secario, P.-T. Ou, A. Alom, J.-J. Liu, and K.-C. Liu, Biosens. Bioelectron. 92, 186 (2017). 10.1016/j.bios.2017.01.043 28214745

[83] V.-T. Nguyen, H. B. Seo, B. C. Kim, S. K. Kim, C.-S. Song, and M. B. Gu, Biosens. Bioelectron. 86, 293 (2016). 10.1016/j.bios.2016.06.064 27387259

[84] J. Xu, D. Suarez, and D. S. Gottfried, Anal. Bioanal. Chem. 389, 1193 (2007). 10.1007/s00216-007-1525-3 17710386

[85] E. Ozkumur, J. W. Needham, D. A. Bergstein, R. Gonzalez, M. Cabodi, J. M. Gershoni, B. B. Goldberg, and M. S. Unlü, Proc. Natl. Acad. Sci. U. S. A. 105, 7988 (2008). 10.1073/pnas.0711421105 18523019 PMC2430348

[86] C. A. Lopez, G. G. Daaboul, R. S. Vedula, E. Özkumur, D. A. Bergstein, T. W. Geisbert, H. E. Fawcett, B. B. Goldberg, J. H. Connor, and M. S. Ünlü, Biosens. Bioelectron. 26, 3432 (2011). 10.1016/j.bios.2011.01.019 21342761 PMC3065545

[87] A. Yurt, G. G. Daaboul, J. H. Connor, B. B. Goldberg, and M. S. Ünlü, Nanoscale 4, 715 (2012). 10.1039/c2nr11562j 22214976 PMC3759154

[88] G. G. Daaboul, C. A. Lopez, J. Chinnala, B. B. Goldberg, J. H. Connor, and M. S. Ünlü, ACS Nano 8, 6047 (2014). 10.1021/nn501312q 24840765 PMC4466106

[89] S. M. Scherr, D. S. Freedman, K. N. Agans, A. Rosca, E. Carter, M. Kuroda, H. E. Fawcett, C. E. Mire, T. W. Geisbert, M. S. Ünlü, and J. H. Connor, Lab Chip 17, 917 (2017). 10.1039/c6lc01528j 28194457

[90] S. M. Scherr, G. G. Daaboul, J. Trueb, D. Sevenler, H. Fawcett, B. Goldberg, J. H. Connor, and M. S. Ünlü, ACS Nano 10, 2827 (2016). 10.1021/acsnano.5b07948 26760677 PMC5019356

[91] A. Y. Ozkumur, F. E. Kanik, J. T. Trueb, C. Yurdakul, and M. S. Ünlü, IEEE J. Sel. Top. Quantum Electron. 25, 1 (2019). 10.1109/jstqe.2018.2854548

[92] P. Singh, Reference Module in Life Sciences (Elsevier, 2017).

[93] C. L. Wong, M. Chua, H. Mittman, L. X. Choo, H. Q. Lim, and M. Olivo, Sensors 17, 2363 (2017). 10.3390/s17102363 PMC5677386 29035344

[94] C. Estmer Nilsson, S. Abbas, M. Bennemo, A. Larsson, M. D. Hämäläinen, and Å. Frostell-Karlsson, Vaccine 28, 759 (2010). 10.1016/j.vaccine.2009.10.070 19857452

[95] S. Fuentes, L. Klenow, H. Golding, and S. Khurana, Sci. Rep. 7, 42428 (2017). 10.1038/srep42428 28186208 PMC5301242

[96] R. Marsh, A. Connor, E. Gias, and G. L. Toms, J. Med. Virol. 79, 829 (2007). 10.1002/jmv.20892 17457900

[97] P. C. Gauger, C. L. Loving, S. Khurana, A. Lorusso, D. R. Perez, M. E. Kehrli, J. A. Roth, H. Golding, and A. L. Vincent, Virology 471-473, 93 (2014). 10.1016/j.virol.2014.10.003 25461535

[98] Health Quality Ontario, Ont. Health Technol. Assess. Ser. 18, 1 (2018). PMC5824029 29487629

[99] Centers for Disease Control and Prevention (U.S.), Antibiotic Resistance Threats in the United States, 2019 (Centers for Disease Control and Prevention (U.S.), 2019).

[100] B. Casini, B. Tuvo, M. L. Cristina, A. M. Spagnolo, M. Totaro, A. Baggiani, and G. P. Privitera, Int. J. Environ. Res. Public Health 16, 3572 (2019). 10.3390/ijerph16193572 PMC6801766 31554297

[101] M. M. Nerandzic, J. L. Cadnum, M. J. Pultz, and C. J. Donskey, BMC Infect. Dis. 10, 197 (2010). 10.1186/1471-2334-10-197 20615229 PMC2910020

[102] B. M. Andersen, in Prevention and Control of Infections in Hospitals: Practice and Theory, edited by Andersen B. M. (Springer International Publishing, Cham, 2019), pp. 815–834.

[103] M. E. R. Darnell, K. Subbarao, S. M. Feinstone, and D. R. Taylor, J. Virol. Methods 121, 85 (2004). 10.1016/j.jviromet.2004.06.006 15350737 PMC7112912

[104] K. Stadler, V. Masignani, M. Eickmann, S. Becker, S. Abrignani, H.-D. Klenk, and R. Rappuoli, Nat. Rev. Microbiol. 1, 209 (2003). 10.1038/nrmicro775 15035025 PMC7097337

[105] A. C. Walls, Y.-J. Park, M. A. Tortorici, A. Wall, A. T. McGuire, and D. Veesler, Cell 181, 281 (2020). 10.1016/j.cell.2020.02.058 32155444 PMC7102599

[106] J. Grossman, A. Pierce, J. Mody, J. Gagne, C. Sykora, S. Sayood, S. Cook, N. Shomer, S. Y. Liang, and S. R. Eckhouse, J. Am. Coll. Surg. 231, 275 (2020). 10.1016/j.jamcollsurg.2020.04.029 32353399 PMC7184977

[107] M. C. Celina, E. Martinez, M. A. Omana, A. Sanchez, D. Wiemann, M. Tezak, and T. R. Dargaville, Polym. Degrad. Stab. 179, 109251 (2020). 10.1016/j.polymdegradstab.2020.109251 32834203 PMC7271865

[108] R. P. Sinha and D.-P. Häder, Photochem. Photobiol. Sci. 1, 225 (2002). 10.1039/b201230h 12661961

[109] J. Koivunen and H. Heinonen-Tanski, Water Res. 39, 1519 (2005). 10.1016/j.watres.2005.01.021 15878023

[110] P. Setlow, Environ. Mol. Mutagen. 38, 97 (2001). 10.1002/em.1058 11746741

[111] N. Nwachuku, C. P. Gerba, A. Oswald, and F. D. Mashadi, Appl. Environ. Microbiol. 71, 5633 (2005). 10.1128/aem.71.9.5633-5636.2005 16151167 PMC1214670

[112] M. Berney, H.-U. Weilenmann, J. Ihssen, C. Bassin, and T. Egli, Appl. Environ. Microbiol. 72, 2586 (2006). 10.1128/aem.72.4.2586-2593.2006 16597961 PMC1449012

[113] W. A. M. Hijnen, E. F. Beerendonk, and G. J. Medema, Water Res. 40, 3 (2006). 10.1016/j.watres.2005.10.030 16386286

[114] K. Bergmann, Am. Pharm. Rev. (2014); available at http://www.americanpharmaceuticalreview.com/Featured-Articles/169257-UV-C-Irradiation-A-New-Viral-Inactivation-Method-for-Biopharmaceuticals/.

[115] A. Guridi, E. Sevillano, I. de la Fuente, E. Mateo, E. Eraso, and G. Quindós, Int. J. Environ. Res. Public Health 16, 4747 (2019). 10.3390/ijerph16234747 PMC6926820 31783593

[116] I. H. Hamzavi, A. B. Lyons, I. Kohli, S. Narla, A. Parks-Miller, J. M. Gelfand, H. W. Lim, and D. M. Ozog, J. Am. Acad. Dermatol. 82, 1511 (2020). 10.1016/j.jaad.2020.03.085 32246972 PMC7214862

[117] S. S. Nunayon, H. Zhang, and A. C. K. Lai, Indoor Air 30, 180 (2020). 10.1111/ina.12619 31688980

[118] X. Li, M. Cai, L. Wang, F. Niu, D. Yang, and G. Zhang, Sci. Total Environ. 659, 1415 (2019). 10.1016/j.scitotenv.2018.12.344 31096352

[119] J. G. B. Derraik, W. A. Anderson, E. A. Connelly, and Y. C. Anderson, medRxiv:2020.04.02.20051409 (2020), 10.1101/2020.04.02.20051409.

[120] A. R. Marra, M. L. Schweizer, and M. B. Edmond, Infect. Control Hosp. Epidemiol. 39, 20 (2018). 10.1017/ice.2017.226 29144223

[121] A. Mustapha, H. Alhmidi, J. L. Cadnum, A. L. Jencson, and C. J. Donskey, Am. J. Infect. Control 46, 584 (2018). 10.1016/j.ajic.2017.10.025 29306489

[122] R. F. Chemaly, S. Simmons, C. Dale, S. S. Ghantoji, M. Rodriguez, J. Gubb, J. Stachowiak, and M. Stibich, Ther. Adv. Infect. Dis. 2, 79 (2014). 10.1177/2049936114543287 25469234 PMC4250270

[123] A. Beal, N. Mahida, K. Staniforth, N. Vaughan, M. Clarke, and T. Boswell, J. Hosp. Infect. 93, 164 (2016). 10.1016/j.jhin.2016.03.016 27107618

[124] L. El Haddad, S. S. Ghantoji, M. Stibich, J. B. Fleming, C. Segal, K. M. Ware, and R. F. Chemaly, BMC Infect. Dis. 17, 672 (2017). 10.1186/s12879-017-2792-z 29017457 PMC5635568

[125] M. M. Nerandzic, P. Thota, T. Sankar C., A. Jencson, J. L. Cadnum, A. J. Ray, R. A. Salata, R. R. Watkins, and C. J. Donskey, Infect. Control Hosp. Epidemiol. 36, 192 (2015). 10.1017/ice.2014.36 25633002

[126] E. Ackerman, IEEE Spectrum: Technol., Eng., Sci. News 57, 50 (2020). 10.1109/mspec.2020.8946313

[127] A. Winfield, Nat. Electron. 2, 46 (2019). 10.1038/s41928-019-0213-6

[128] A. Jobin, M. Ienca, and E. Vayena, Nat. Mach. Intell. 1, 389 (2019). 10.1038/s42256-019-0088-2

[129] R. Dias and A. Torkamani, Genome Med. 11, 70 (2019). 10.1186/s13073-019-0689-8 31744524 PMC6865045

